# Evolution of China’s industry–university–research–hospital collaboration in AI medical devices and implications for medical institutions and public health governance

**DOI:** 10.3389/fpubh.2026.1852483

**Published:** 2026-06-10

**Authors:** Feng Hu, Huijie Yang, Zhimin Ren, Xiaoping Wang, Haiyan Zhou, Shaobo Yang, Shaobin Wei, Jiahan Hu, Shuang Zhao, Hao Hu, Junyu Cheng

**Affiliations:** 1 Institute of International Business & Economics Innovation and Governance, Shanghai University of International Business and Economics, Shanghai, China; 2 Industrial Technology Research Center, Shanghai Yice Research Institute, Shanghai, China; 3 International Business School, Shanghai University of International Business and Economics, Shanghai, China; 4 Institutional Affiliation School of Management, Zhejiang Gongshang University Hangzhou College of Commerce, Hangzhou, China; 5 College of Management, Ningbo University of Finance & Economics, Ningbo, China; 6 Graduate School, Nueva Ecija University of Science and Technology, Cabanatuan, Philippines; 7 College of Engineering, University of Perpetual Help System Laguna, Laguna, Philippines; 8 School of Economics, Shanghai University, Shanghai, China; 9 Elliott School of International Affairs, The George Washington University, Washington, DC, United States

**Keywords:** artificial intelligence medical devices, digital health, industry–university–research–hospital collaboration, medical institutions, social network analysis

## Abstract

Against the backdrop of China’s rapid expansion of artificial intelligence (AI), and intelligent healthcare policies, the integration of AI into medical devices is reshaping healthcare delivery and innovation systems. Based on patent data on industry–university–research cooperation in the China AI medical device industry from 2019 to 2025, this study adopts social network analysis and geographical detector methods to systematically examine the spatiotemporal evolution and influencing factors of the innovation network across three dimensions. The findings reveal that at the microagent level, the early dominant pattern of enterprises has been gradually reconstructed by the participation of universities, research institutes, and medical institutions, with medical institutions rising from marginal participants to core intermediaries. At the provincial level, the network exhibits prominent unbalanced agglomeration characteristics. Beijing’s status as a national hub has been continuously strengthened, and a multipolar spatial pattern has taken initial shape. At the level of the cooperation type, intraprovincial cooperation is dominated by industry–industry collaboration, while interprovincial cooperation has formed a cross-regional synergy framework with Beijing as the core, radiating to the Yangtze River Delta and the Pearl River Delta. The value added of the financial industry, the volume of import and export trade, and the transaction volume of the technology market are revealed as the core factors explaining the network status of provinces, whereas the explanatory power of the proportion of education expenditure remains weak. This study contributes to a deeper understanding of China’s AI medical device industry and offers practical insights regarding public health governance.

## 1 Introduction

In recent years, the deep integration of artificial intelligence (AI) and the medical device industry has become a strategic focus of global technological competition and transformation in the health care field (1, 2). As a typical representative of new productive forces, AI medical devices, i.e., medical devices that realize medical applications through AI technologies based on “medical device data,” are reshaping the service models of disease diagnosis, treatment, and health management via high-precision perception, AI algorithm-assisted decision-making, and data-driven clinical support.^1^ To accelerate the application of AI in the healthcare industry, multiple Chinese government departments have successively issued a series of supportive and standardized policies, such as the Implementation Opinions on the Special Action for “AI + Manufacturing” and the Implementation Opinions on Promoting and Regulating the Application and Development of “AI + Medical and Health Care.” In terms of market scale, the AI medical device industry is entering a period of rapid growth. According to statistics from research institutes, the global AI medical device market size is projected to increase from USD 18.8 billion in 2025 to USD 133.2 billion in 2032, with a compound annual growth rate reaching 32.3%.^2^ However, the innovative development of the AI medical device industry requires in-depth collaboration among multiple stakeholders, including industry, universities, research institutes, and hospitals (39–41). As a country with a huge demand for medical resources, China urgently needs to boost the development of this industry through innovation, which is crucial to national health governance.

Existing research on AI medical devices has focused mainly on two aspects. The first is the compliance with laws and regulations for AI medical devices, their ability to improve safety performance, and supervision and medical liability. For instance, Putera et al. adopted systematic legal reasoning methods and reported deficiencies in the current medical device conformity assessment in addressing AI changes (3). Niemiec argued that the Medical Device Regulation (MDR) might help improve the safety and performance of medical AI devices in the European market, yet the impact of this regulation depends on its full implementation by EU member states (4). The second aspect is the systematic study of the current technological innovation trends of the industry through patent analysis of AI medical devices. For example, Benjamens et al. analyzed patents in this industry based on the Espacenet database and reported rapid growth in industry patents, with Chinese patents accounting for more than 60% of the total (5). Zhang et al. conducted a patent analysis of AI medical devices and revealed that major global medical device giants such as Philips and Siemens occupied leading positions in the global frontier of technological innovation, while Chinese universities and research institutes, including Zhejiang University, Tianjin University, and the Shenzhen Institute of Advanced Technology, had outstanding performance in technological innovation (6).

In research on innovation networks, scholars generally adopt social network analysis (SNA). Based on patent application and transfer data, paper cooperation, project cooperation, and other sources, they conduct research on the node status, network attributes, structural holes, and influencing factors in innovation networks at spatial scales such as the national, regional, provincial, and urban levels (7–16). Existing studies on industry–university–research (IUR) collaborative innovation networks, which are an important aspect of research on innovation networks, have focused mainly on the spatiotemporal evolutionary characteristics and influencing factors of such networks (17–20). For example, Wang et al. studied the IUR collaborative innovation network of low-carbon technologies in China. They reported that the intensity of IUR collaborative innovation in southern China was higher than that in northern China. In contrast, the collaborative innovation intensity between universities and research institutes was relatively weak (21). Pu et al. conducted a study on IUR cooperation in the field of lithium battery energy storage technology based on patent cooperation data and reported that the network nodes were closely connected and that state-owned organizations and institutions held prominent node positions (22). Peng et al. applied the exponential random graph model (ERGM) to study the IUR cooperation patents of China’s Double First-Class universities and revealed that the depth and breadth of knowledge, as well as the organizational capabilities of IUR cooperation parties, played important roles in network formation (23). Sun et al. researched the IUR collaborative innovation network within the Chengdu–Chongqing urban agglomeration and reported that government support had a significant promoting effect on both the urban network status and the overall network connection intensity (24). Li et al. analyzed the IUR collaborative innovation network of the biomedical industry in Fujian Province. They reported that the main types of innovative cooperation were university–industry, industry–industry, and IUR cooperation, with universities playing a core hub role in the network (25).

A review of the literature reveals several aspects that need to be improved. First, there are relatively few studies on multiscale IUR collaborative innovation networks in terms of network construction. Second, from the perspective of research subjects, there is a lack of research on IUR cooperation models from the microsubject perspective. Third, from the perspective of industry focus, research on emerging industries such as AI medical devices is insufficient. Fourth, in the field of medical device technological innovation, medical institutions such as hospitals and health centers play important roles in innovation networks, and their cooperation models with industry, universities, and research institutes constitute important paths for collaborative innovation; however, few existing IUR studies have incorporated medical institutions into innovation networks. In 2019, the Center for Medical Device Evaluation of the National Medical Products Administration took the lead in establishing the “AI Medical Device Innovation Cooperation Platform”, integrating the forces of the government, industry, universities, research institutes, and end-users. This marked the official incorporation of medical institutions into the national-level innovation collaboration system. Since then, clinical institutions such as Peking Union Medical College Hospital and Peking University Third Hospital have begun to participate extensively in patent cooperation, becoming key nodes connecting basic research and clinical application. These evidences indicate that medical institutions have gradually become key players in the R&D of this industry in China. Accordingly, based on the IUR cooperation patent data of China’s AI medical device industry from 2019 to 2025, this paper adopts social network analysis and geographical detector methods (Geodetetctor) to systematically examine the spatiotemporal evolutionary characteristics and influencing factors of the innovation network from three dimensions: patent applicants, provincial regions and types of industry–university–research–hospital (IURH) cooperation. This study further discusses the practical implications for medical institutions to participate in innovation networks and enhance their innovation capabilities, aiming to seize the development opportunities of the AI medical device industry and optimize the allocation of innovative resources.

The innovations of this paper are as follows. First, the three-dimensional analysis helps reveal the spatial pattern of innovative resources and the cooperation models among innovative subjects in the AI medical device industry. Second, addressing the insufficient attention paid to the role of medical institutions in existing research, this paper incorporates Chinese medical institutions as independent subjects into the analysis. By systematically examining indicators such as weighted degree centrality, betweenness centrality, and cooperation intensity, this study provides empirical evidence for understanding the functional positioning of medical institutions in innovation networks. Lastly, this study contributes to the digital health literature by demonstrating how innovation networks facilitate the translation of technology into public health benefits.

## 2 Research data and methods

### 2.1 Research objects and data

The data used in this paper are derived from the Incopat Global Patent Database. The data retrieval and processing steps are as follows: First, a total of 17,242 patents were retrieved from the database with the knowledge-intensive classification, 502 medical device manufacturing, the emerging industry classification 1.5 artificial intelligence, more than one patent applicant, and China as the publication country. Second, through data screening and sorting, 6,685 relevant patents from 2019 to 2025 were ultimately obtained. Third, for patent applicant cooperation, if there were three applicants (e.g., A, B, and C), it was defined as one cooperation between A and B, one between A and C, and one between B and C. This method was used to construct the IUR collaborative innovation network of patent applicants in the AI medical device industry. Fourth, the Qixin Huiyan Enterprise Database was used to query the provincial locations of patent applicants in batches, from which the IUR collaborative innovation network of provincial regions in the AI medical device industry was constructed. Fifth, according to the IURH classification, stakeholders were categorized as follows: industry includes enterprises and other industrial entities; universities include universities and graduate schools; research institutes include academies and research institutes; and hospitals include general hospitals and affiliated hospitals (26–28). Due to data limitations, this paper does not distinguish between entities engaged in innovation activities and the patent applicants.

### 2.2 Research methods

1. Social network analysis

Using Gephi software, we constructed innovation networks for IURH collaboration among patent applicants in the AI medical device industry and among provinces by year. In networks, nodes represent applicants and edges represent collaboration among applicants. We then calculated the weighted degree centrality and betweenness centrality of provinces and patent applicants without standardization (5, 6, 26). Among these, weighted degree centrality reflects the intensity of direct collaboration between a target node and other nodes within the network. A higher value indicates that the innovation entities have established closer cooperative relationships and engage in more frequent collaboration within the collaborative network (34–38).


$$\mathrm{WD}C_{i}=\sum_{j=1}^{N}w_{\mathrm{ij}}$$

$w_{\mathrm{ij}}$ represents the weight of the edge between node i and node j . *i* represents the applicant or the province and is used to calculate the weighted degree centrality of the patent applicant and the province, respectively.

Betweenness centrality characterizes the intermediary bridging function of nodes in the network. Nodes with high betweenness centrality occupy pivotal connecting positions and facilitate knowledge flow and resource integration.


$$BC_{i}=\sum_{i\neq s\neq t}\frac{\sigma_{\mathrm{st}}(i)}{\sigma_{\mathrm{st}.}}$$

$\sigma_{\mathrm{st}}(i)$ represents the number of shortest paths passing through node i . $\sigma_{\mathrm{st}}$ represents the total number of shortest paths between node s and node t .

1. Geodetector

A comprehensive review of previous studies reveals that IURH collaboration in AI medical devices is closely associated with regional economic development, scientific and technological innovation, and local consumption levels (9–20). Accordingly, this paper takes the provincial weighted degree centrality and betweenness centrality of the IURH collaborative innovation network as the dependent variables (29–33).

From the dimension of economic development and the level of openness, GDP, per capita GDP, the value added of the financial industry, and the import and export trade volume are selected as explanatory variables. From the dimension of residents’ income and consumption level, urban per capita disposable income and total retail sales of social consumer goods are chosen as explanatory variables. From the dimension of scientific and technological innovation input and output, the technology market transaction volume, the proportion of education expenditure, the proportion of scientific expenditure, and the R&D expenditure of industrial enterprises above a designated size are selected as explanatory variables. Before conducting factor detection with the Geodetector, ArcGIS is used to classify all variables into 5 grades via the natural breaks method. The explanatory variable data are derived from the China Statistical Yearbook. Since the statistical data for 2025 have not yet been updated, only the influencing factors from 2019 to 2024 are analyzed in this paper. The range of *q* is 0~1 . The larger the value of *q*, the stronger the explanatory power of that driving factor for the spatial differentiation characteristics of the collaborative innovation network.

## 3 Results and analysis

### 3.1 Network structural characteristics from the perspective of patent applicants

From the perspective of the weighted degree centrality of patent applicants, the IURH collaborative innovation network in the field of AI medical devices has shown a trend of subject diversification. In 2019, the top-ranked applicants were dominated by enterprises such as Jiangsu Yuyue Medical Equipment and the BOE Technology Group Co., Ltd. However, in 2021, the centrality of universities, including Tsinghua University and Shandong University, as well as medical institutions such as Shandong Cancer Hospital, increased markedly. In particular, Tsinghua University ranked first, indicating that academic research forces have been deeply integrated into the IURH cooperation network of AI medical devices. Meanwhile, clinical demands have been incorporated into the source of innovation, and the integration of technological research and development with medical scenarios has gradually become a core driving force for network evolution. As an institutional innovation initiative, the Innovation Cooperation Platform for AI Medical Devices, led by the Medical Device Evaluation Center of the National Medical Products Administration, has not only provided institutional support for its rising status in the network but also broken down information barriers and eased restrictions on the flow of academic resources. In 2023, the network structure became further complex. Universities such as Tsinghua University, Beihang University, and Fudan University maintained high centrality rankings, while the inclusion of clinical institutions such as Peking University Third Hospital and Jiangsu Provincial People’s Hospital reflected the continuous enhancement of the status of medical institutions in the network. In 2025, leading consumer electronics and home appliance enterprises such as Haier Smart Home and Gree Electric Appliances (Zhuhai) entered the network with strong momentum, alongside the participation of cross-industry entities such as China Mobile and Taikang Insurance. This pattern may be associated with both technological spillovers driven by industrial upgrading and further policy incentives. Specifically, seven government departments, including the Ministry of Industry and Information Technology, jointly issued the Implementation Plan for the Digital and Intelligent Transformation of the Pharmaceutical Industry (2025–2030). This policy vigorously promotes cross-industry integrated development and facilitates the intelligent upgrading of the pharmaceutical industry across the entire industrial chain by providing policy endorsement and coordinated resource support, covering research and development, production, operation, and other key links. The involvement of these entities has also helped promote the widespread adoption of inclusive public health services, such as home health monitoring and smart management of chronic diseases (Table 1).

**Table 1:** Weighted degree centrality of patent applicants in the IURH collaborative innovation network of the AI medical device industry (top 15).

Rank	Patent applicant	2019	Patent applicant	2021	Patent applicant	2023	Patent applicant	2025
1	Jiangsu Yuyue Information Systems Co., Ltd.	22	Tsinghua University	70	Tsinghua University	41	Haier Smart Home Co., Ltd.	200
2	BOE Technology Group Co., Ltd.	20	Suzhou Yuyue Medical Technology Co., Ltd.	42	Inventec (Shanghai) Technology Co., Ltd.	40	Gree Electric Appliances, Inc. of Zhuhai	188
3	Shenzhen E-Techco Information Technology Co., Ltd.	20	Shandong University	40	Neusoft Medical Systems Co., Ltd.	38	Zhuhai Lianyun Technology Co., Ltd.	188
4	Shenzhen Qianhai Anke Information Technology Co., Ltd.	20	State Grid Corporation of China	36	Guangdong Power Grid Co., Ltd.	37	China Mobile Communications Group Co., Ltd.	152
5	State Grid Corporation of China	17	Shandong Cancer Institute (Shandong Cancer Hospital)	27	Inventec (Shanghai) Electronics Co., Ltd.	32	Guangzhou Shiyuan Electronic Technology Co., Ltd.	109
6	China Mobile Communications Group Co., Ltd.	17	Zhejiang Tsinghua Flexible Electronics Technology Research Institute	26	Shanghai Neusoft Medical Technology Co., Ltd.	31	Tsinghua University	101
7	Suzhou Medical Supplies Factory Co., Ltd.	16	Tianjin Kaixin Life Technology Co., Ltd.	23	Electric Power Research Institute of Guangdong Power Grid Co., Ltd.	31	Taikang Insurance Group Co., Ltd.	83
8	Suzhou Hermiz Health Technology Co., Ltd.	13	Tianjin New Kaixin Life Technology Co., Ltd.	23	Beijing University of Aeronautics and Astronautics	30	Shenzhen Mindray Bio-Medical Electronics Co., Ltd.	78
9	Suzhou Mithyssel Artificial Intelligence Co., Ltd.	13	Beijing Yikang Medical Technology Co., Ltd.	21	China Mobile Communications Group Co., Ltd.	28	Guangzhou Xike Medical Device Technology Co., Ltd.	74
10	Nanjing Yuyue Software Technology Co., Ltd.	13	Jinan Bishan Network Technology Co., Ltd.	21	South China University of Technology	28	Qingdao Haier Technology Co., Ltd.	74
11	Research Institute of China Mobile Communications Co., Ltd.	13	Unisound Intelligent Technology Co., Ltd.	20	Fudan University	28	Qingdao Haier Intelligent Technology R&D Co., Ltd.	73
12	General Hospital of the Chinese People’s Liberation Army (PLA)	13	Xiamen Unisound Intelligent Technology Co., Ltd.	20	Zhejiang University	27	Guangzhou Shiyuan Artificial Intelligence Innovation Research Institute Co., Ltd.	73
13	China General Nuclear Power Group	11	Suzhou Medical Supplies Factory Co., Ltd.	20	Nanjing University of Posts and Telecommunications	27	Taikang Pension Co., Ltd.	72
14	Jiangsu Yuyue Medical Equipment Co., Ltd.	11	Peking Union Medical College Hospital, Chinese Academy of Medical Sciences	20	Peking University Third Hospital (Peking University Third Clinical Medical College)	26	China Mobile (Chengdu) Information and Communication Technology Co., Ltd.	71
15	Daya Bay Nuclear Power Operation and Management Co., Ltd.	10	China Petroleum & Chemical Corporation	18	Migu Culture Technology Co., Ltd.	23	Jiangsu Yuyue Kailite Biotechnology Co., Ltd.	71
					Aitang (Suzhou) Electronic Technology Co., Ltd.			
					Jiangsu Provincial People’s Hospital (Nanjing Medical University First Affiliated Hospital)			

From the perspective of the betweenness centrality of patent applicants (Table 2), State Grid Corporation of China ranked first, with a betweenness centrality of 217 in 2019, and most of the top-ranked applicants were the State Grid and its affiliated institutions. In 2021, Tsinghua University surged to the top, with a betweenness centrality of 2,234, and the betweenness centrality of universities and research institutes such as Peking University, the University of Electronic Science and Technology of China, and the Aerospace Information Research Institute of the Chinese Academy of Sciences had markedly increased. This finding indicates that academic research forces had begun to assume the bridging role of connecting various stakeholders in the IURH collaborative innovation network for AI medical devices. In this phase, clinical institutions, including Peking Union Medical College Hospital, Chinese Academy of Medical Sciences, and the Chinese PLA General Hospital, entered the rankings for the first time. In 2023, Peking Union Medical College Hospital, Chinese Academy of Medical Sciences, took first place, with a betweenness centrality of 17,279.67, closely followed by Tsinghua University. Clinical institutions such as the Cancer Hospital, Chinese Academy of Medical Sciences, Peking University Third Hospital, and Peking University First Hospital emerged collectively, with their betweenness centrality values far exceeding those of any previous period. This finding indicates that medical institutions had evolved from mere cooperative participants to core intermediaries in the network, undertaking the pivotal function of linking basic research, technological R&D, and clinical application. In 2025, the betweenness centrality values of network nodes soared further, forming a dual-core pattern centered on Peking University Third Hospital and Peking Union Medical College Hospital, Chinese Academy of Medical Sciences. Universities and research institutes, including the Shenzhen Institute of Advanced Technology of the Chinese Academy of Sciences and Tsinghua University, constituted a closely connected second echelon for IUR cooperation in this field.

**Table 2:** Betweenness centrality of patent applicants in the industry-academia-research collaboration innovation network for the AI medical device industry (top 15).

Rank	Patent applicant	2019	Patent applicant	2021	Patent applicant	2023	Patent applicant	2025
1	State Grid Corporation of China	217	Tsinghua University	2,234	Peking Union Medical College Hospital, Chinese Academy of Medical Sciences	17,279.66667	Peking University Third Hospital (The Third Clinical Medical College of Peking University)	179,009.9578
2	Beijing China Power Puhua Information Technology Co., Ltd.	40	Peking University	1825	Tsinghua University	17,251.83333	Peking Union Medical College Hospital, Chinese Academy of Medical Sciences	176,831.0012
3	Anhui Nari Jiyuan Power Grid Technology Co., Ltd.	40	University of Electronic Science and Technology of Chi	1,554	Cancer Hospital, Chinese Academy of Medical Sciences	11,988	Peking Union Medical College Hospital, Chinese Academy of Medical Sciences	125,190.432
4	Northeastern University	38	Aerospace Information Research Institute, Chinese Academy of Sciences	1,483	Beijing Institute of Technology	8,420	Tsinghua University	111,581.3597
5	Shenzhen University	26	Beijing University of Aeronautics and Astronautics	1,256	Peking University Third Hospital (The Third Clinical Medical College of Peking University)	7,875	Tianjin University	87,110.58626
6	State Grid Information & Communication Industry Group Co., Ltd.	21	State Grid Corporation of China	674	Tianjin University	5,942	Shanghai Jiao Tong University	84,393.88631
7	Disaster Prevention and Mitigation Center, State Grid Hunan Electric Power Co., Ltd.	21	Shandong University	661	Wenzhou Institute of Safety (Emergency) Technology, Tianjin University	5,916	Sun Yat-sen University	64,622.06928
8	Electric Power Research Institute, State Grid Shandong Electric Power Co., Ltd.	21	Peking Union Medical College Hospital, Chinese Academy of Medical Sciences	575	Tencent Technology (Shenzhen) Co., Ltd.	5,407	Beijing Jiakang Zhongzhi Technology Co., Ltd	64,576.29943
9	East China Tianhuangping Pumped Storage Co., Ltd.	21	Shenzhen Mindray Bio-Medical Electronics Co., Ltd.	446	Sun Yat-sen University	5,135	Shandong University	63,710.24458
10	Beijing University of Aeronautics and Astronautics	20	General Hospital of the Chinese People’s Liberation Army (PLA)	306	Beijing Jiakang Zhongzhi Technology Co., Ltd	3,932.166667	Fudan University	59,491.10849
11	Tsinghua University	18	Beijing Teeview Technology Co., Ltd.	300	Peking University First Hospital	3,898.333333	Zhejiang University	58,184.57183
12	Huashan Hospital Fudan University	16	Beijing Luhe Hospital Affiliated to Capital Medical University	300	Beijing University of Aeronautics and Astronautics	3,579	Beijing Tiantan Hospital, Affiliated with Capital Medical University	56,440.78617
13	CERNET Network Co., Ltd.	16	Institute of Agricultural Information, Chinese Academy of Agricultural Sciences	300	Dalian University of Technology	3,346	Beijing University of Aeronautics and Astronautics	53,488.70815
14	BOE Technology Group Co., Ltd.	15	Huludao Power Supply Company, State Grid Liaoning Electric Power Co., Ltd.	270	China University of Geosciences (Wuhan)	3,270	Beijing University of Posts and Telecommunications	52,564.61797
15	General Hospital of the Chinese People’s Liberation Army	10	State Grid Liaoning Electric Power Co., Ltd.	248	South China University of Technology	3,128	Huashan Hospital Fudan University	52,489.65361

From the perspective of cooperation intensity among patent applicants (Table 3), IURH cooperation in AI medical devices in 2019 was dominated by collaborations between the internal subsidiaries of enterprises, which reveals that the industry lacked a multi-stakeholder coordination mechanism in its early stages, with Shenzhen E-Techco and Shenzhen Qianhai Ance Testing ranking first with a cooperation intensity of 20. Notably, the embryonic stage of a small number of IURH collaborations emerged during this period, such as the partnership between Chengdu True Dimension Technology and the Affiliated Zhongshan Hospital of Dalian University and the collaboration between the Chinese PLA General Hospital and Beijing Hanbo Information Technology Co., Ltd. Although the cooperation intensity was generally low, it indicates that medical institutions had begun to establish initial IUR cooperation pathways with universities and enterprises. These features are in line with the focus of industrial policies in this period, namely the construction of basic supporting facilities, the initial improvement of regulatory systems, and the establishment of interactive platforms for diverse participants.^3^

Cooperation in 2021 exhibited a prominent feature of deepened IURH synergy. While the internal corporate collaboration between Tianjin Kaixin Life Technology and Tianjin New Kaixin Life Technology still took the top spot, the consortium of the Zhejiang Tsinghua Flexible Electronics Technology Research Institute and Tsinghua University tied for first place, with a cooperation intensity of 23. The Shandong Cancer Institute and Shandong University entered the top ranks for the first time, with an intensity of 13, and Peking Union Medical College Hospital of the Chinese Academy of Medical Sciences also made the list with Shenzhen Mindray Bio-Medical Electronics Co., Ltd., at an intensity of 11. Internal corporate collaborative innovation remained important in this phase. In 2023, the internal corporate cooperation between Shanghai Neusoft Medical Technology and Neusoft Medical Systems Co., Ltd., ranked first, with a cooperation intensity of 31. Jiangsu Provincial People’s Hospital and Nanjing University of Posts and Telecommunications entered the top 15 with an intensity of 12, becoming a typical case of in-depth synergy between medical institutions and universities. During this stage, relevant policies shifted from encouraging general applications to focusing on scenario-driven development.^4^ The participation of medical institutions helps bridge the gap between academic research and practical application, and accelerates the marketization of targeted R&D achievements. Against such a policy background, the categories of IURH cooperation have become more diversified, and the cooperation intensity has increased steadily. In 2025, Gree Electric Appliances, Inc., of Zhuhai and Zhuhai Lianyun Technology took a dominant lead by a wide margin, with a cooperation intensity of 188, followed by the Taikang Insurance Group, Inc., and Taikang Pension, with 72. Consortia such as China Mobile (Chengdu) Information and Communication Technology and China Mobile Communications, Qingdao Haier Intelligent Technology R&D and Haier Smart Home, as well as Shenzhen Mindray Bio-Medical Electronics and Shenzhen Mindray Technology, closely trailed behind. This finding demonstrates that industry giants in home appliances, insurance, telecommunications, and other sectors are actively entering the AI medical device track through internal collaborative innovation, with their cooperation intensity far surpassing that of previous IUR consortia.

**Table 3:** Collaboration intensity among patent applicants in the industry-academia-research innovation network for the AI medical device industry (top 15).

Rank	Applicant 1	Applicant 2	2019	Applicant 1	Applicant 2	2021	Applicant 1	Applicant 2	2023	Applicant 1	Applicant 2	2025
1	Shenzhen E-Techco Information Technology Co., Ltd.	Shenzhen Qianhai Anke Information Technology Co., Ltd.	20	Tianjin Kaixin Life Technology Co., Ltd.	Tianjin New Kaixin Life Technology Co., Lt	23	Shanghai Neusoft Medical Technology Co., Ltd.	Neusoft Medical Systems Co., Ltd.	31	Gree Electric Appliances Inc. of Zhuhai	Zhuhai Lianyun Technology Co., Ltd.	188
2	Jiangsu Yuyue Information System Co., Ltd.	Nanjing Yuyue Software Technology Co., Ltd.	13	Zhejiang Tsinghua Flexible Electronics Technology Research Institute	Tsinghua University	23	Inventec (Shanghai) Technology Co., Ltd.	Inventec (Shanghai) Electronics Co., Ltd.	28	Taikang Insurance Group Co., Ltd.	Taikang Pension Insurance Co., Ltd.	72
3	Research Institute of China Mobile Communications Co., Ltd.	China Mobile Communications Group Corporation	13	Unisound Intelligent Technology Co., Ltd.	Xiamen Unisound Intelligent Technology Co., Ltd.	20	Guangdong Power Grid Co., Ltd.	Guangdong Power Grid Co., Ltd. Electric Power Science Research Institute	28	China Mobile (Chengdu) Information and Communication Technology Co., Ltd.	China Mobile Communications Group Corporation	68
4	Jiangsu Yuyue Medical Equipment Co., Ltd.	Suzhou Medical Supplies Factory Co., Ltd.	11	Jiangsu Yuyue Medical Equipment Co., Ltd.	Suzhou Yuyue Medical Technology Co., Ltd.	14	BOE Technology Group Co., Ltd.	Beijing BOE Display Technology Co., Ltd.	18	Qingdao Haier Intelligent Technology R&D Co., Ltd.	Haier Smart Home Co., Ltd.	67
5	Suzhou Hermiz Health Technology Co., Ltd.	Suzhou Mithyssel Artificial Intelligence Co., Ltd.	9	China Petroleum & Chemical Corporation	Sinopec Qingdao Safety Engineering Institute	13	Migu Cultural Technology Co., Ltd.	China Mobile Communications Group Corporation	18	Shenzhen Mindray Bio-Medical Electronics Co., Ltd.	Shenzhen Mindray Technology Co., Ltd.	66
6	Chengdu True Dimension Technology Co., Ltd.	The Affiliated Zhongshan Hospital of Dalian University	9	Shandong Cancer Prevention and Treatment Institute (Shandong Cancer Hospital)	Shandong University	13	Hangzhou Weiming Xinke Technology Co., Ltd.	Peking University Information Technology Institute (Hangzhou)	17	Guangzhou CVTE Electronic Technology Co., Ltd.	Guangzhou Xike Medical Device Technology Co., Ltd.	55
7	Hefei BOE Optoelectronics Technology Co., Ltd.	BOE Technology Group Co., Ltd.	8	Shandong Zhengxin Medical Technology Co., Ltd.	Jiangsu Zhengxin Intelligent Technology Co., Ltd.	12	Fudan University	Zhuhai Fudan Innovation Research Institute	17	Guangzhou CVTE Electronic Technology Co., Ltd.	Guangzhou CVTE Artificial Intelligence Innovation Research Institute Co., Ltd.	53
8	The General Hospital of the Chinese People’s Liberation Army	Beijing Hanbo Information Technology Co., Ltd.	8	Nanjing Yidu Cloud Medical Technology Co., Ltd.	Nanjing Yiyun Big Data Technology Co., Ltd.	11	Shenzhen Institute of Advanced Technology	Shenzhen Technology University of the Chinese Academy of Sciences (Preparatory)	16	Qingdao Haier Technology Co., Ltd.	Haier Smart Home Co., Ltd.	47
9	Tianjin Hairen Medical Technology Co., Ltd.	Tianjin Hengyu Medical Technology Co., Ltd.	7	Jiangsu Yuyue Information System Co., Ltd.	Suzhou Yuyue Medical Technology Co., Ltd.	11	Zhongke Borui (Beijing) Technology Co., Ltd.	Zhongke Bokang (Beijing) Medical Equipment Co., Ltd.	15	Research Institute of China Mobile Communications Co., Ltd.	China Mobile Communications Group Corporation	35
10	Jiadong Health Technology (Wuhu) Co., Ltd.	Jiadong Health Technology (Wuhu) Co., Ltd.	6	Peking Union Medical College Hospital, Chinese Academy of Medical Sciences	Shenzhen Mindray Bio-Medical Electronics Co., Ltd.	11	Beijing Airdoc Technology Co., Ltd.	Shanghai Airdoc Medical Technology Co., Ltd.	15	Sun Yat-sen University·Shenzhen	Sun Yat-sen University	35
11	Hunan Shunhong Intelligent Technology Co., Ltd.	Changsha Military-Civil Advanced Technology Research Co., Ltd.	6	LinkDoc Technology (Beijing) Co., Ltd.	LinkDoc Technology (Tianjin) Co., Ltd.	10	South China University of Technology	Guangdong Artificial Intelligence and Digital Economy Laboratory (Guangzhou)	14	Jiangsu Yuyue Kailite Biotechnology Co., Ltd.	Jiangsu Yuekai Biotechnology Co., Ltd.	33
12	Shenzhen Dedao Health Management Co., Ltd.	Beijing Yuntianyuan Technology Co., Ltd.	6	Migu Interactive Entertainment Co., Ltd.	Migu Culture Technology Co., Ltd.	9	Qilu University of Technology (Shandong Academy of Sciences)	Shandong Computer Science Center (National Supercomputer Center in Jinan)	13	Jiangsu Yuyue Kailite Biotechnology Co., Ltd.	Zhejiang Kailite Medical Device Co., Ltd.	31
13	BOE Optoelectronics Technology Co., Ltd.	BOE Technology Group Co., Ltd.	5	Suzhou Yuyue Medical Technology Co., Ltd.	Suzhou Medical Supplies Factory Co., Ltd.	9	Jiangsu Provincial People’s Hospital (Nanjing Medical University First Affiliated Hospital)	Nanjing University of Posts and Telecommunications	12	Haier U + Smart Technology (Beijing) Co., Ltd.	Haier Smart Home Co., Ltd.	28
14	Suzhou Medical Supplies Factory Co., Ltd.	Jiangsu Yuyue Information System Co., Ltd.	5	Tsinghua University	Beijing Pins Medical Equipment Co., Ltd.	9	Zhejiang Kailite Medical Device Co., Ltd.	Jiangsu Yuekai Biotechnology Co., Ltd.	11	Hangzhou Deepwise Blolink Technology Co., Ltd.	Beijing Deepwise Blolink Technology Co., Ltd.	28
15	Suzhou Hermiz Health Technology Co., Ltd.	Henan Butianshi Technology Co., Ltd.	4	Chongqing Nanpeng AI Industry Research Institute Co., Ltd.	Guangzhou Tianpeng Computer Technology Co., Ltd.	8	China Energy Bioenergy Power Generation Group Co., Ltd.	North China Electric Power University	11			
	Suzhou Mithyssel Artificial Intelligence Co., Ltd.	Henan Butianshi Technology Co., Ltd.	4	Nanjing Yuyue Software Technology Co., Ltd.	Suzhou Yuyue Medical Technology Co., Ltd.	8	Beijing University of Aeronautics and Astronautics Hangzhou Innovation Institute	Beijing University of Aeronautics and Astronautics	11			
	Guangdong Nuclear Power Joint Venture Co., Ltd.	China General Nuclear Power Group Co., Ltd.	4	Shanghai Zhiqing Medical Technology Co., Ltd.	Eye & ENT Hospital of Fudan University	8						
	Suzhou Yuyue Medical Technology Co., Ltd.	Jiangsu Yuyue Information System Co., Ltd.	4	Capital Medical University	China Academy of Electronics and Information Technology	8						
	Shandong Normal University	Jinan Baleson Instrument Co., Ltd.	4									
	China Mobile (Hangzhou) Information Technology Co., Ltd.	China Mobile Communications Group Corporation	4									
	Nanjing Renkang Hospital Co., Ltd.	Shenyang Shenbei Shenyi Hospital	4									
	Duke Kunshan University	The Third Affiliated Hospital of Sun Yat-sen University (Sun Yat-sen University Hepatobiliary Hospital)	4									
	China Astronaut Research and Training Center	Beijing Institute of Technology	4									
	Beijing Tongren Hospital Affiliated to Capital Medical University	Beijing University of Aeronautics and Astronautics	4									

### 3.2 Network structural characteristics from the provincial perspective

From the perspective of provincial weighted degree centrality (Table 4), the IURH collaborative innovation network for AI medical devices exhibits a prominent feature of unbalanced agglomeration. In 2019, Guangdong ranked first, with a weighted degree centrality of 266, closely followed by Beijing and Jiangsu. This reflects the fact that, supported by the province’s strong manufacturing base, digital economy, and smart hardware industry, enterprise-led collaboration has dominated the early stages. These three provinces jointly formed the first echelon of the collaborative innovation network for IUR cooperation, while eastern provinces, including Shanghai, Zhejiang, and Anhui, followed in the rankings, presenting an obvious pattern of coastal agglomeration in the overall network structure. In 2021, Beijing surged to the top with a weighted degree centrality of 634, with Jiangsu and Guangdong taking second and third places, respectively, and Shandong rose to fourth place with a value of 245, indicating the continuous improvement in innovation vitality in the Bohai Rim region.

In 2023, Beijing took a substantial lead with a weighted degree centrality of 891, followed by Guangdong (728) and Jiangsu (471). Shanghai, Zhejiang, and Shandong constituted the second echelon, and the rankings of central and western provinces such as Sichuan, Liaoning, and Shaanxi rose steadily. Notably, Beijing’s leading edge further expanded in this phase, reflecting that the national capital is accelerating its development into a national hub for AI medical device innovation under the superimposed effects of its concentrated high-quality resources, including top universities, research institutes, leading enterprises, and clinical medical resources. In 2025, Beijing achieved a dominant lead, with a weighted degree centrality of 2,326, and Guangdong closely followed with 2,079. Jiangsu, Shandong, Shanghai, and Zhejiang formed a closely coordinated second echelon, with their weighted degree centrality values all ranging from 900 to 1,100. The weighted degree centrality of provinces such as Sichuan, Anhui, Hubei, Tianjin, and Shaanxi also generally exceeded 200, which demonstrates the continuous expansion of the scale of the IURH collaborative innovation network for AI medical devices and the initial formation of a multipolar spatial pattern.

In addition, Shanghai has consistently remained in the second echelon. The underlying reason can be interpreted from the analysis of patent applicants above: Beijing is home to top-tier universities such as Tsinghua University and Peking University, as well as world-class clinical institutions such as Peking Union Medical College Hospital of the Chinese Academy of Medical Sciences and the Chinese PLA General Hospital. Furthermore, Beijing continues to allocate policy resources to this industry, with a commitment to establishing the city as a hub of innovation where AI and the pharmaceutical industry converge.^5^ Guangdong has forged a close IUR synergy between leading enterprises such as Shenzhen Mindray and Guangzhou CVTE and prestigious universities, including Sun Yat-sen University and South China University of Technology. Jiangsu has built a dense innovation network by integrating local enterprises such as Suzhou Yuyue and Nanjing Medbrain Cloud with provincial universities. In contrast, while Shanghai has a large number of innovation entities, its patent output is relatively dispersed and lacks a dominant core hub, resulting in a clustering effect driven by weighted degree centrality that falls short of that seen in the former.

**Table 4:** Provincial weighted degree centrality in the IURH collaborative innovation network of the AI medical device industry (top 15).

Rank	Province	2019	Province	2021	Province	2023	Province	2025
1	Guangdong	266	Beijing	634	Beijing	891	Beijing	2,326
2	Beijing	254	Jiangsu	428	Guangdong	728	Guangdong	2,079
3	Jiangsu	203	Guangdong	342	Jiangsu	471	Jiangsu	1,038
4	Shanghai	99	Shandong	245	Shanghai	460	Shandong	984
5	Zhejiang	63	Shanghai	207	Zhejiang	295	Shanghai	928
6	Anhui	52	Zhejiang	192	Shandong	252	Zhejiang	897
7	Shandong	51	Tianjin	116	Sichuan	149	Sichuan	435
8	Tianjin	36	Henan	73	Liaoning	134	Anhui	395
9	Hubei	34	Sichuan	65	Shaanxi	120	Hubei	329
10	Liaoning	28	Liaoning	59	Hubei	107	Tianjin	290
11	Hunan	25	Chongqing	55	Tianjin	92	Shaanxi	234
12	Sichuan	25	Hubei	53	Chongqing	89	Henan	205
13	Henan	20	Fujian	50	Anhui	87	Fujian	170
14	Fujian	17	Hunan	48	Fujian	76	Chongqing	167
15	Chongqing	12	Anhui	44	Henan	76	Hebei	156

From the perspective of provincial betweenness centrality (Table 5), Beijing ranked first, with a betweenness centrality of 0.39, in 2019, closely followed by Anhui, Shandong, Jiangsu, and Guangdong, yet the values remained generally low across all provinces. In 2021, the betweenness centrality values experienced rapid growth: the value for Beijing increased to 144.86; those for Guangdong and Jiangsu reached 89.57 and 55.92, respectively; and the values for Shanghai, Zhejiang, Shandong, Sichuan, Shaanxi, and other provinces also rose markedly. Leveraging its concentrated cluster of top universities, research institutes, leading enterprises, and world-class clinical resources, Beijing rapidly emerged as a national innovation hub, undertaking the core function of connecting major regions across the country.

In 2023, Beijing’s betweenness centrality slightly decreased to 129.32 but still maintained an absolute leading position, while the values of Guangdong, Shaanxi, Jiangsu, Zhejiang, Shandong, Sichuan, and other provinces presented a more balanced distribution pattern. Shaanxi rose to third place with a betweenness centrality of 22.08, and central and western provinces, including Chongqing, Heilongjiang, and Guizhou, entered the rankings for the first time. This finding reflects that the hub function of the IURH collaborative innovation network for AI medical devices is evolving from a single-core structure to a polycentric pattern. Notably, although Beijing still had a high betweenness centrality value, its relative monopolistic position in the national network weakened compared with that in 2021, and provinces such as Guangdong, Jiangsu, and Shaanxi began to assume the role of regional subhubs.

In 2025, Beijing’s betweenness centrality further dropped to 63.67 but still firmly held the top rank. Jiangsu surged to second place with 26.45, and Guangdong ranked third at 18.57. Shaanxi, Shanghai, Sichuan, Guangxi, Zhejiang, Henan, Shandong and other provinces formed a closely coordinated second echelon. This pattern indicates that the national innovation network is shifting from single-polar driving to multipolar synergy and that the roles played by various provinces in knowledge flow have become more diversified. Although there have been policies to empower the healthcare industry across the country, integrating with AI, provincial responses to these policies have yielded divergent results. For example, the AI healthcare industry policies of both Shanghai and Suzhou can be categorized into four stages that are emergence, layout, integration, boom, and the progression of these policy phases has been highly synchronized. However, Shanghai has consistently maintained a relatively high weighted degree centrality, reflecting a high concentration of direct inter-organizational cooperation. In contrast, Jiangsu has demonstrated a more pronounced intermediary role, serving as a stronger bridge. This difference may be related to the distinct regional development models of the two provinces. Shanghai primarily functions as a hub for high-end healthcare and technological innovation, while Jiangsu relies more heavily on its integrated manufacturing system and the broader Yangtze River Delta (YRD) innovation network to drive the diffusion of collaboration and cross-regional connectivity. This trend toward multipolarity has broken the pattern of one-way flow of innovation resources, enabling provinces in central and western China to gain more convenient access to advanced medical technologies through cooperation with regional sub-centers, thereby effectively alleviating regional imbalances in public health resources.

**Table 5:** Provincial betweenness centrality in the IURH collaborative innovation network of the AI medical device industry (top 15).

Rank	Province	2019	Province	2021	Province	2023	Province	2025
1	Beijing	0.39	Beijing	144.86	Beijing	129.32	Beijing	63.67
2	Anhui	0.21	Guangdong	89.57	Guangdong	68.61	Jiangsu	26.45
3	Shandong	0.13	Jiangsu	55.92	Shaanxi	22.08	Guangdong	18.57
4	Jiangsu	0.10	Shanghai	22.18	Jiangsu	16.73	Shaanxi	15.88
5	Guangdong	0.10	Zhejiang	15.46	Zhejiang	14.01	Shanghai	15.19
6	Shanghai	0.09	Shandong	15.01	Shandong	11.42	Sichuan	13.80
7	Hebei	0.04	Sichuan	12.05	Sichuan	10.53	Guangxi	11.46
8	Zhejiang	0.03	Shaanxi	11.52	Shanghai	10.04	Zhejiang	8.90
9	Liaoning	0.02	Hunan	8.24	Chongqing	9.76	Henan	8.07
10	Shaanxi	0.00	Henan	3.63	Heilongjiang	9.33	Shandong	7.62
11	Shanxi	0.00	Guangxi	3.36	Guizhou	8.84	Hebei	6.24
12	Tianjin	0.00	Tianjin	2.85	Henan	7.42	Chongqing	5.96
13	Sichuan	0.00	Liaoning	2.64	Hubei	4.44	Hubei	5.42
14			Anhui	1.25	Liaoning	4.04	Xinjiang	3.42
15			Hubei	0.80	Jiangxi	2.06	Tianjin	2.87

**Figure 1:**
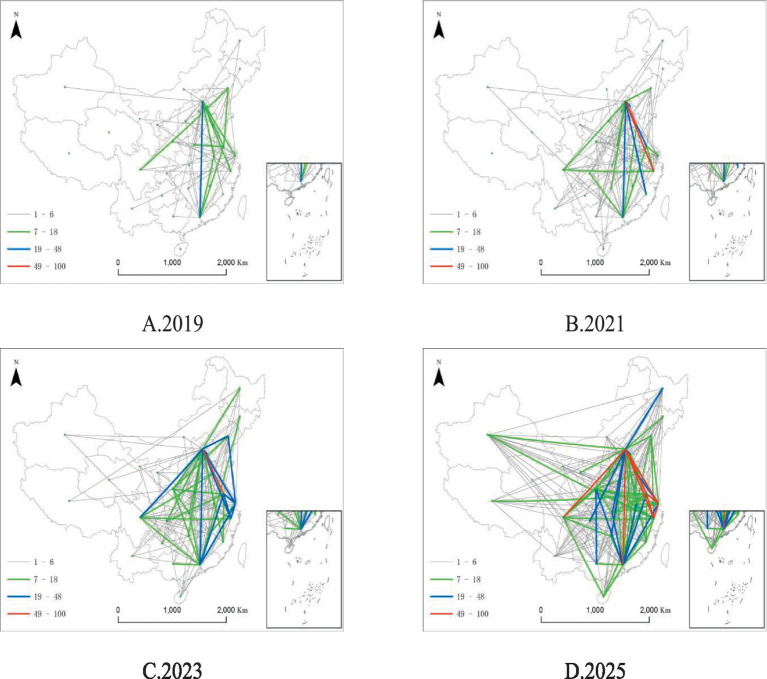
Provincial IURH collaborative innovation network of the AI medical device industry. **(A)** 2019; **(B)** 2021; **(C)** 2023; **(D)** 2025.

From the perspective of the innovation intensity. In 2019, the collaboration network was dominated by low-intensity connections. Cooperative innovation among adjacent provinces, such as Beijing and Hebei, Jiangsu and Anhui, took the lead, and no high-intensity cross-regional collaboration had yet formed. This reflected that early IUR cooperative innovation activities in AI medical devices were highly constrained by geographical proximity and administrative boundaries. By 2021, the collaboration intensity of core regional pairs, including Beijing–Hebei and Jiangsu–Zhejiang, had increased significantly, with some connections rising to the medium-to-high intensity level. This indicated an accelerated pace of innovation integration within urban agglomerations such as the Beijing-Tianjin-Hebei region (BTH) and the YRD. Meanwhile, the emergence of cross-regional IUR cooperative innovation, such as between Beijing and Guangdong, Shanghai and Sichuan, marked the initial formation of a national collaboration network. In 2023, the IUR cooperative innovation intensity of key provincial pairs, Beijing–Hebei, Jiangsu–Zhejiang, and Guangdong–Beijing, fell into the medium-to-high range of 19 to 48. Notably, the collaboration between Beijing–Jiangsu and Beijing–Guangdong neared the threshold of high intensity. Additionally, the intensity of cross-regional IUR cooperation between the central-western and eastern coastal provinces, such as Shandong–Beijing, Sichuan–Guangdong, saw a remarkable rise. By 2025, the collaboration network entered a stage of high integration. Jiangsu and Zhejiang ranked first with a collaboration intensity of 100, and core pairs including Beijing–Hebei, Beijing–Jiangsu, and Guangdong–Beijing reached the high-intensity range of 49 to 100. IUR cooperative innovation within the three major urban agglomerations, YRD, BTH, Pearl River Delta, hit an unprecedented level, and the interconnection among the three agglomerations fully entered the medium-to-high intensity interval, which would not have been possible without the impetus provided by regional integration policies, including the integration of the YRD and the Greater Bay Area. Provinces such as Anhui, Shandong, Sichuan, and Hubei witnessed a steady increase in their IUR collaboration intensity with core hub provinces. Even remote provinces, including Xinjiang, Guangxi, and Heilongjiang, began to form low-intensity connections with core regions, demonstrating the continuous expansion of the coverage of the IUR cooperative innovation network for AI medical devices (Figure 1).

### 3.3 Characteristics of IURH collaborative innovation from a spatial dimension

In terms of the types of intraprovincial IURH cooperation (Table 6), the collaborative innovation models for AI medical devices have presented an increasingly diversified pattern, with the depth and breadth of cooperation improving in tandem. Industry–industry cooperation has remained the dominant type, and the intensity of internal corporate collaborative innovation in provinces such as Guangdong, Jiangsu, Beijing, and Shandong has risen steadily. Industry–university cooperation has grown rapidly, with the intensity of collaborative innovation between universities and enterprises rising markedly in Jiangsu, Beijing, Shandong, Zhejiang, and other provinces. This enables local clinical needs to be rapidly translated into innovative outcomes, thereby enhancing the responsiveness and precision of regional public health services.

With respect to industry–hospital cooperation, Beijing took the lead in such cooperation in 2019, closely followed by Guangdong and Shanghai; however, the overall cooperation intensity was limited. In 2025, Beijing had a substantial lead with a cooperation intensity of 100; Shanghai and Guangdong tied for second place with 71 each, and provinces, including Shandong and Zhejiang, also entered the top ranks. With respect to university–hospital cooperation, Guangdong, Beijing, and Jiangsu were the main participants in 2019, but the cooperation intensity was generally low across the board. In 2025, Beijing achieved an absolute leading position with a cooperation intensity of 124; Shanghai ranked second with 68; Guangdong ranked third with 56; and Jiangsu and Shandong closely followed.

In terms of hospital–research cooperation, provinces such as Beijing, Guangdong, Shanghai, and Anhui have consistently ranked toward the top. The overall intensity of hospital–hospital cooperation has remained limited, with Guangdong, Beijing, and Shanghai maintaining the top rankings in this type of cooperation.

**Table 6:** Intraprovincial IURH cooperation types in the AI medical device industry (top 5).

Type	2019	2021	2023	2025
Industry–industry	Guangdong (62), Jiangsu (50), Beijing (20), Shanghai (15), Tianjin (11), Zhejiang (11)	Jiangsu (97), Beijing (39), Tianjin (31), Guangdong (28), Zhejiang (20)	Guangdong (111), Beijing (105), Shanghai (51), Jiangsu (41), Shandong (23)	Guangdong (505), Beijing (250), Shandong (226), Jiangsu (151), Zhejiang (65)
Industry–research	Beijing (7), Guangdong (6), Tianjin (4), Shandong (3), Jiangsu (2)	Guangdong (18), Beijing (12), Shanghai (11), Zhejiang (8), Jiangsu (6)	Guangdong (31), Zhejiang (26), Shanghai (14), Jiangsu (10), Beijing (7)	Guangdong (31), Zhejiang (30), Beijing (22), Shandong (20), Shanghai (13)
Industry–hospital	Beijing (16), Guangdong (6), Shanghai (5), Jiangsu (2), Henan (1), Hunan (1), Zhejiang (1)	Shanghai (29), Beijing (27), Guangdong (16), Jiangsu (11), Shandong (8)	Beijing (44), Shanghai (19), Guangdong (16), Jiangsu (16), Shandong (8), Zhejiang (8)	Beijing (100), Shanghai (71), Guangdong (71), Shandong (38), Zhejiang (29)
University–university	Beijing (4), Guangdong (3), Heilongjiang (1), Hubei (1), Jilin (1), Jiangsu (1), Inner Mongolia (1), Sichuan (1)	Guangdong (7), Beijing (4), Hubei (3), Hunan (3), Shanghai (3)	Sichuan (11), Shanxi (11), Shanghai (8), Shaanxi (7), Zhejiang (6)	Guangdong (42), Beijing (30), Sichuan (12)
University–research	Beijing (9), Hubei (2), Jiangsu (2), Shandong (1), Shanghai (1)	Beijing (16), Shanghai (5), Guangdong (5), Henan (2), Jiangsu (1), Shandong (1), Guangxi (1), Jilin (1), Shaanxi (1)	Guangdong (38), Beijing (29), Shandong (23), Zhejiang (12), Anhui (5)	Zhejiang (47), Beijing (44), Hubei (31), Shandong (22), Shanghai (22)
University–hospital	Guangdong (7), Beijing (6), Jiangsu (4), Shanghai (2), Henan (1), Shandong (1), Sichuan (1), Chongqing (1)	Beijing (27), Shandong (15), Guangdong (10), Jiangsu (5), Shanghai (5)	Beijing (29), Guangdong (25), Jiangsu (22), Shanghai (17), Chongqing (8)	Beijing (124), Shanghai (68), Guangdong (56)
Research–research	Anhui (2), Beijing (2), Hainan (1)	Beijing (4), Shanghai (2), Zhejiang (2), Guangdong (1)	Guangdong (7), Shanghai (4), Anhui (1), Fujian (1)	Jiangsu (48), Shandong (34)
Hospital–research	Guangdong (8), Beijing (4), Chongqing (3), Shanghai (2), Jiangsu (1)	Guangdong (14), Beijing (4), Jiangsu (1), Anhui (1), Hebei (1)	Beijing (6), Guangdong (3), Jiangsu (2), Zhejiang (2), Chongqing (1)	Beijing (11), Guangdong (10), Henan (6), Anhui (5), Shanghai (2)
Hospital–hospital	Guangdong (6), Shandong (1)	Henan (2), Anhui (1), Hunan (1), Tianjin (1)	Beijing (5), Guangdong (3), Guizhou (3)	Beijing (38), Guangdong (22), Shanghai (15), Anhui (12), Jiangsu (9), Zhejiang (9)
Industry–university	Jiangsu (12), Shandong (10), Zhejiang (9), Hubei (8), Shanghai (8)	Jiangsu (28), Shandong (25), Beijing (25), Zhejiang (16), Guangdong (16)	Shanghai (3), Guangxi (2)	Guangdong (20), Beijing (10), Shanghai (10), Shandong (6), Chongqing (4), Zhejiang (4)

In terms of interprovincial IURH cooperation types, Beijing (Table 7), as a national innovation hub, occupies a core position in almost all cooperation types, and its IUR collaborative innovation with Jiangsu, Zhejiang, Shandong, Guangdong, Shanghai, and other provinces forms the backbone of the cross-regional cooperation network.

With respect to industry–industry cooperation, the Beijing–Jiangsu partnership ranks first with a cooperation intensity of 182, closely followed by the Beijing–Zhejiang, Beijing–Shandong, Jiangsu–Zhejiang and Shanghai–Jiangsu partnerships. These findings indicate that the industrial synergy of AI medical devices between the BTH and the YRD, the two core regions, has become the main channel for cross-regional cooperation. Notably, the Beijing–Sichuan collaboration enters the top ranks with an intensity of 103, reflecting the accelerated formation of industrial linkages in AI medical devices between the Chengdu–Chongqing region and the BTH. This trend demonstrates that eastern and western regions are capable of collaborating on major national public health issues, such as major infectious diseases and rare diseases, thereby significantly enhancing the overall resilience of the national public health system.

In terms of industry–university cooperation, Beijing–Jiangsu takes the lead, with a cooperation intensity of 42, and partnerships including Shanghai–Beijing, Beijing–Guangdong, Beijing–Guangdong, Beijing–Hunan and Beijing–Zhejiang form a closely connected second echelon. This finding reveals an increasingly frequent knowledge flow of AI medical devices between top universities and industrially strong provinces.

With respect to industry–hospital cooperation, Beijing–Guangdong has a substantial lead with a cooperation intensity of 84, and Shanghai–Jiangsu ranks second with 47, followed by Beijing–Zhejiang, Shanghai–Beijing, Beijing–Jiangsu, Beijing–Heilongjiang and other combinations. Notably, Beijing–Heilongjiang enters the top ranks with an intensity of 28, which demonstrates that the high-quality medical resources and innovative elements of AI medical devices in the old industrial base of Northeast China are forming new collaborative innovation pathways.

In terms of university–hospital cooperation, Beijing–Zhejiang leads with a cooperation intensity of 23, closely followed by Beijing–Heilongjiang with 21, and combinations such as Guangdong–Jiangsu, Beijing–Guangdong, and Beijing–Tianjin also make the top ranks. With respect to hospital–research cooperation, Beijing–Guangdong ranks first with a cooperation intensity of 14, and partnerships including Shanghai–Guangdong, Beijing–Zhejiang, Shanghai–Beijing, and Guangdong–Henan form a diversified collaborative innovation network. The core policy context underlying these cross-provincial, multi-faceted IURH collaborations is the convergence of *the national innovation-driven development strategy*, *the Healthy China initiative*, and *the “AI+” strategy*. Leveraging specialized policies and top-tier resources, Beijing has emerged as a hub radiating these diverse forms of collaboration. Integration policies for the BTH and the YRD have established the main framework for cross-regional cooperation, driving high-intensity cross-regional coordination. Meanwhile, policies such as *the Western Development Strategy*, *the Chengdu-Chongqing Economic Circle*, and *the Revitalization of Northeast China* have helped the integration of regions like Sichuan and Heilongjiang with the BTH, allowing new pathways for cooperation and sharing resources. Based on these diverse policies, the cross-regional cooperation landscape is showing a pattern of nationwide synergy and balanced development.

**Table 7:** Interprovincial IURH cooperation types in the AI medical device industry (top 10).

Type	2019–2025
Industry–industry	Beijing–Jiangsu (182), Beijing–Zhejiang (159), Beijing–Shandong (144), Jiangsu–Zhejiang (132), Shanghai–Jiangsu (129), Beijing–Guangdong (123), Beijing–Sichuan (103), Shanghai–Beijing (99), Guangdong–Jiangsu (76), Shanghai–Guangdong (69)
Industry–university	Beijing–Jiangsu (42), Shanghai–Beijing (35), Beijing–Guangdong (29), Beijing–Hunan (28), Beijing–Zhejiang (24), Beijing–Shandong (23), Jiangsu–Zhejiang (21), Beijing–Anhui (20), Shanghai–Jiangsu (19), Beijing–Sichuan (16), Shandong–Guangdong (16)
Industry–research	Beijing–Guangdong (30), Beijing–Jiangsu (24), Guangxi–Shaanxi (21), Beijing–Zhejiang (19), Beijing–Shandong (15), Shanghai–Zhejiang (13), Shanghai–Beijing (11), Shandong–Jiangsu (11), Shanghai–Guangdong (11), Guangdong–Zhejiang (8), Sichuan–Tianjin (8)
Industry–hospital	Beijing–Guangdong (84), Shanghai–Jiangsu (47), Beijing–Zhejiang (43), Shanghai–Beijing (34), Beijing–Jiangsu (28), Beijing–Heilongjiang (28), Shanghai–Guangdong (26), Guangdong–Jiangsu (24), Beijing–Shandong (19), Shanghai–Zhejiang (19)
University–university	Beijing–Henan (18), Jilin–Guangdong (16), Shanghai–Beijing (15), Beijing–Shaanxi (10), Beijing–Tianjin (9), Hubei–Shaanxi (8), Beijing–Hubei (7), Beijing–Hebei (7), Beijing–Liaoning (7), Beijing–Sichuan (7)
University–research	Beijing–Zhejiang (37), Shanghai–Guangdong (31), Beijing–Guangdong (21), Shanghai–Zhejiang (15), Shanghai–Beijing (13), Beijing–Jiangsu (13), Guangdong–Shaanxi (12), Beijing–Tianjin (11), Beijing–Anhui (10), Tianjin–Guangdong (9), Guangdong–Hubei (9), Sichuan–Guangdong (9)
University–hospital	Beijing–Zhejiang (23), Beijing–Heilongjiang (21), Guangdong–Jiangsu (16), Beijing–Guangdong (15), Beijing–Tianjin (14), Shanghai–Beijing (11), Beijing–Liaoning (11), Shanghai–Liaoning (9), Beijing–Hubei (6), Beijing–Shandong (6), Shanghai–Jiangsu (6)
Research–research	Beijing–Guangdong (18), Shanghai–Guangdong (6), Beijing–Jiangsu (5), Shanghai–Zhejiang (5), Beijing–Tianjin (4), Shanghai–Beijing (4), Beijing–Zhejiang (3), Guangdong–Zhejiang (3), Beijing–Guangxi (3), Henan–Zhejiang (3)
Hospital–research	Beijing–Guangdong (14), Shanghai–Guangdong (12), Beijing–Zhejiang (12), Shanghai–Beijing (10), Guangdong–Henan (10), Shanghai–Jiangsu (8), Jiangsu–Zhejiang (7), Beijing–Hubei (6), Guangdong–Hubei (5), Beijing–Jiangsu (4), Shanghai–Zhejiang (4), Guangdong–Guangxi (4), Beijing–Heilongjiang (4)
Hospital–hospital	Beijing–Shandong (6), Beijing–Zhejiang (5), Shandong–Jiangsu (4), Beijing–Guangdong (3), Beijing–Hubei (3), Yunnan–Beijing (3), Sichuan–Chongqing (3), Beijing–Hebei (3), Inner Mongolia–Beijing (3), Shanghai–Yunnan (3)

## 4 Analysis of network influencing factors

Analysis of the correlation between the weighted degree centrality of the IURH collaborative innovation network in the AI medical device industry and its influencing factors yields the following findings (Table 8).

From the dimension of economic development and the level of openness, the value added of the financial industry exhibits sustained and strong explanatory power, with its *Q* values remaining at a high level throughout the 2019–2024 period and all passing the significance test. This finding indicates that the concentration of financial resources is among the most critical variables determining the status of provinces in the innovation network, by improving the supply of technology through the allocation of R&D funds and the development of medical infrastructure, thereby enhancing the province’s capacity to address public health issues. The import and export trade volume also performs prominently, with its *Q* values ranking among the top of all factors in most years. A higher level of openness facilitates access to cutting-edge international technologies, medical equipment, and innovative resources, accelerates technological innovation and advancement, and enhances the quality of science and technology services for public health. Although the explanatory power of GDP and per capita GDP is slightly lower than that of the two factors above, it remains stable overall, demonstrating that the total economic volume and development quality are important supports for the provincial innovation network status, and it must not be overlooked when advancing AI in healthcare.

From the dimension of residents’ income and consumption level, the explanatory power of urban per capita disposable income is approximately 0.6 and passes the significance test in all years, indicating that the residents’ income level exerts a stable explanatory effect on the provincial innovation network status, and driving demand for healthcare services can spur the advancement of medical technology. The explanatory power of the total retail sales of social consumer goods shows obvious fluctuations and fails to pass the significance test in some years, which implies that the impact of the consumer market scale on innovation may have phased characteristics and can become an important driving force in specific periods. However, overall, its role in health governance under cross-regional technical collaboration is limited.

From the dimension of scientific and technological innovation input and output, the technology market transaction volume delivers the most outstanding performance, with its *Q* values remaining high and significant in all years during the 2019–2024 period, making it one of the core driving factors with the strongest explanatory power in the weighted degree centrality analysis. From the perspective of public health governance, the development of the technology market not only helps promote the diffusion of key technologies and the commercialization of research outcomes, but also accelerates the application of AI in healthcare systems, thereby improving the efficiency and quality of regional public healthcare. The proportion of scientific expenditure also performs steadily and passes the significance test in all years, reflecting that enhancing public health governance capabilities through technological breakthroughs is inseparable from government investment in scientific and technological resources. The explanatory power of the R&D expenditure of industrial enterprises above a designated size is significant in most years, indicating that enterprise R&D investment is also a key factor affecting the provincial innovation network status. As companies become more innovative, AI can be integrated more quickly into primary healthcare systems, further driving the digital transformation of public health governance. In contrast, the proportion of education expenditure shows extremely weak performance and fails to pass the significance test in all years. The reason for this result may be that the scale and structure of education funds have not been effectively transformed into innovative advantages.

**Table 8:** Influencing factors of provincial weighted degree centrality in the IURH collaborative innovation network of the AI medical device industry.

Influencing dimension	Influencing factor	2024		2023		2022		2021		2020		2019	
		*Q* value	*p* value	*Q* value	*p* value	*Q* value	*p* value	*Q* value	*p* value	*Q* value	*p* value	*Q* value	*p* value
Economic development and level of openness	GDP	0.508	0.051	0.632	0.007	0.598	0.013	0.643	0.005	0.823	0.000	0.627	0.007
	GDP per capita	0.650	0.004	0.624	0.008	0.522	0.036	0.620	0.007	0.579	0.020	0.485	0.059
	Value added of the financial sector	0.822	0.000	0.882	0.000	0.688	0.002	0.795	0.000	0.792	0.000	0.871	0.000
	Total import and export value	0.837	0.000	0.867	0.000	0.866	0.000	0.770	0.000	0.655	0.011	0.751	0.004
Residents’ income and consumption level	Urban per capita disposable income	0.601	0.021	0.723	0.000	0.567	0.034	0.676	0.004	0.653	0.005	0.618	0.008
	Total retail sales of social consumer goods	0.517	0.012	0.628	0.000	0.466	0.105	0.536	0.045	0.373	0.202	0.683	0.000
Scientific and technological innovation input and output	Technology market transaction volume	0.583	0.074	0.694	0.004	0.795	0.000	0.745	0.004	0.865	0.000	0.826	0.000
	Share of education expenditure	0.267	0.216	0.190	0.457	0.200	0.410	0.098	0.680	0.109	0.667	0.070	0.770
	Share of science and technology expenditure	0.569	0.006	0.600	0.003	0.565	0.006	0.726	0.000	0.519	0.009	0.831	0.000
	R&D expenditure of industrial enterprises above a designated size	0.510	0.049	0.654	0.004	0.530	0.043	0.631	0.007	0.403	0.147	0.582	0.019

Analysis of betweenness centrality and its influencing factors in the IURH cooperation network of the AI medical device industry reveals the following findings (Table 9). From the dimension of economic development and the level of openness, the value added of the financial industry shows a pattern that is similar to that in the weighted degree centrality analysis, passing the significance test in most years. This finding indicates that the concentration of financial resources not only affects a province’s status in the innovation network but also shapes its intermediary function in the network at a deeper level. The explanatory power of the import and export trade volume exhibits a fluctuating upward trend and is significant in some years, which suggests that the impact of the level of openness on the structural position in the network may have phased characteristics. If there is excessive reliance on such nodes, this would be highly susceptible to fluctuations in the international political and economic environment, making it difficult to establish a stable cross-regional public health support mechanism. The explanatory power of GDP and per capita GDP is significantly weaker than that in the weighted degree centrality analysis and fails to pass the significance test in most years, indicating that the total economic volume and development quality have a weaker explanatory effect on the network’s intermediary function than innovation function. This suggests that, in the absence of proactive resource sharing and the design of collaborative mechanisms, economically developed regions may exacerbate the innovation siphon effect, thereby further widening the gap in public health capacity across regions.

From the dimension of residents’ income and consumption level, the explanatory power of all indicators declines markedly compared with that in the weighted degree centrality analysis. Urban per capita disposable income passes the significance test only until 2021, and the total retail sales of social consumer goods pass the test only in 2019. This pattern demonstrates that residents’ income and consumption levels have little effect on a province’s hub status in the innovation network in the later stage of network development. When considering weighted centrality, it becomes clear that market mechanisms alone cannot resolve the issue of R&D imbalance across the country. Underdeveloped regions struggle to generate effective demand for high-end smart medical technologies, making it difficult for them to attract innovation resources through market demand. Ultimately, this leads to a widening gap in technology governance capabilities.

From the dimension of scientific and technological innovation input and output, all indicators show obvious phased characteristics in their performance. The technology market transaction volume reached a peak in 2021 and then dropped rapidly, reflecting that the development level of the technology market exerts a notable effect on a province’s hub status in the network. The proportion of scientific expenditure is significant in most years, indicating that government investment in science and technology plays an important role in influencing betweenness centrality. The overall explanatory power of the R&D expenditure of industrial enterprises above a designated size and the proportion of education expenditure is limited. The latter fails to pass the significance test in all years, showing a negligible explanatory effect on shaping the network’s hub function.

The fact that the proportion of education expenditure fails to pass the significance test in all years for both weighted degree centrality and betweenness centrality merits an in-depth analysis. The proportion of education expenditure reflects the input level of basic education, which has a weak direct correlation with higher education and the cultivation of research-oriented talent. In contrast, innovation in the AI medical device field relies more on high-level research universities and clinical research hospitals, whose funding sources are often independent of local fiscal education expenditure. In addition, from the perspective of the time lag between talent cultivation and innovation output, the impact of education input on innovation is characterized by a significant lag and indirectness. This indicates that relevant departments need to make strategic plans in advance to provide a solid talent base for the development of the public health system.

**Table 9:** Influencing factors of provincial betweenness centrality in the IURH collaborative Innovation Network of the AI medical device industry.

Influencing dimension	Influencing factor	2024		2023		2022		2021		2020		2019	
		*Q* value	*p* value	*Q* value	*p* value	*Q* value	*p* value	*Q* value	*p* value	*Q* value	*p* value	*Q* value	*p* value
Economic development and level of openness	GDP	0.557	0.008	0.341	0.255	0.225	0.310	0.461	0.081	0.348	0.117	0.392	0.090
	GDP per capita	0.350	0.234	0.338	0.253	0.550	0.028	0.418	0.126	0.694	0.003	0.438	0.122
	Value added of the financial sector	0.470	0.125	0.539	0.098	0.317	0.346	0.582	0.047	0.476	0.082	0.504	0.100
	Total import and export value	0.570	0.030	0.587	0.036	0.386	0.159	0.675	0.006	0.648	0.003	0.640	0.007
Residents’ income and consumption level	Urban per capita disposable income	0.423	0.125	0.377	0.213	0.435	0.159	0.489	0.068	0.713	0.000	0.510	0.055
	Total retail sales of social consumer goods	0.322	0.109	0.340	0.110	0.142	0.570	0.398	0.168	0.201	0.438	0.477	0.035
Scientific and technological innovation input and output	Technology market transaction volume	0.547	0.081	0.602	0.071	0.590	0.100	0.846	0.000	0.672	0.030	0.617	0.060
	Share of education expenditure	0.098	0.663	0.096	0.711	0.080	0.728	0.096	0.675	0.062	0.830	0.036	0.928
	Share of science and technology expenditure	0.408	0.059	0.317	0.160	0.357	0.106	0.506	0.008	0.507	0.010	0.646	0.004
	R&D expenditure of industrial enterprises above a designated size	0.388	0.108	0.439	0.096	0.206	0.375	0.467	0.075	0.220	0.384	0.319	0.236

## 5 Conclusions and implications

### 5.1 Conclusion

Based on a systematic analysis of the IURH collaborative innovation network in the AI medical device industry, this paper draws the following key conclusions:

1. The structure of innovative subjects in the collaborative innovation network of the AI medical device industry has undergone a profound transformation from enterprise-led to multistakeholder synergy. At the patent applicant level, universities and research institutes rose rapidly after 2021, becoming bridges connecting various innovative subjects. Medical institutions emerged collectively from 2023, with the betweenness centrality of clinical institutions surging significantly, forming a dual-core hub pattern.
2. The provincial innovation network presents a prominent pattern of coexisting unbalanced agglomeration and multipolarization. Beijing has maintained the top position for a long time, with its leading edge continuously expanding. Guangdong, Jiangsu, Shandong, Shanghai, and Zhejiang form a closely coordinated second echelon, and the rankings of central and western provinces have risen steadily. Beijing’s betweenness centrality slightly declined after peaking at 144.86 in 2021, and provinces including Jiangsu, Guangdong, and Shaanxi have begun to assume the functions of regional subhubs, indicating that the national innovation network is shifting from single-polar driving to multipolar synergy.
3. Regarding IURH cooperation types, intraprovincial collaboration is dominated by industry-industry partnerships, with internal corporate innovation intensity rising steadily in Guangdong, Jiangsu, Beijing and other provinces. Cross-sector collaboration has grown rapidly, with Beijing holding an absolute lead in industry-hospital and university-hospital cooperation. Interprovincially, a cross-regional synergy framework centered on Beijing and radiating nationwide has taken shape. Top cooperation paths span multiple types including industry-industry, industry-university and industry-hospital, and collaborative innovation among the three major urban agglomerations has entered a high-intensity stage.
4. Factor analysis reveals that for weighted degree centrality, the value added of the financial industry, import–export trade volume, technology market transaction volume, and proportion of scientific expenditure are all statistically significant and key determinants of provincial network status. Urban per capita disposable income also shows stable explanatory power, while the proportion of education expenditure remains insignificant across all years. For betweenness centrality, the value added of the financial industry still exhibits strong explanatory power. Technology market transaction volume, though declining after peaking in 2020–2021, remains significant. The proportion of scientific expenditure played an important role in the early stage, whereas the explanatory power of residents’ income and consumption indicators decreased markedly.

### 5.2 Practical implications and recommendations

1. The results of this study reveal that the betweenness centrality of leading medical institutions has continued to increase and that these institutions have become key nodes connecting basic research, technological R&D, and clinical application. For medical institutions, exercising hub function not only facilitates resource acquisition but also guides innovation to meet public health needs. Therefore, leading hospitals should strengthen hub awareness, establish dedicated IURH cooperation offices, and coordinate clinical demand exploration, network expansion, and achievement translation to improve disease diagnosis and public health service capacity.
2. The continuous improvement in industry–hospital cooperation intensity indicates that collaborative innovation between enterprises and medical institutions has become an important path for technological transformation. Thus, medical institutions should establish and improve a regular cooperation mechanism with enterprises and explore the coconstruction of joint laboratories and clinical trial centers with leading enterprises to shorten the cycle of innovative achievement transformation from the laboratory to the bedside, and expand access to high-quality smart healthcare services for the general public.
3. Geodetector analysis shows that regions with concentrated resources, high openness, and active technology transactions, such as Beijing, Shanghai, and Guangdong, should fully leverage their locational advantages to lead cross-regional resource integration and promote the popularization of advanced medical technologies. Hospitals in central and western provinces should actively connect with high-quality resources in eastern regions to narrow regional public health gaps and improve the overall equity of national public health services.

### 5.3 Theoretical contributions

1. This study expands the analytical framework for innovation network research. Previous studies have focused mostly on the analysis of network structure at a single level. This paper combines the microsubject and provincial perspectives and introduces the subdivided dimension of IURH cooperation types to construct a multilevel and multitype analytical framework. This framework can not only depict the evolutionary characteristics of the innovation network more comprehensively but also provide a reference methodological basis for subsequent research.
2. This study enriches the theoretical discussion on the evolution of innovative spatial patterns. Starting from the dual paths of intraprovincial agglomeration and cross-regional radiation, this paper systematically depicts the spatial allocation characteristics of innovative resources in the AI medical device industry. These findings about the status of cities and provinces verify the dynamic evolutionary trend of the core–periphery structure in innovation geography.

### 5.4 Research limitations and prospects

1. There are limitations in data coverage. IURH cooperation takes various forms, and patent cooperation is only one of them. Nonpatent forms of cooperation, such as technology contracts, joint laboratories, joint talent training, and the co-publication of papers and monographs, are not included in the analysis, which may affect the presentation of network integrity. In addition, the adopted data are restricted to patents registered in China, failing to capture the impacts of overseas-related factors, such as Sino-foreign cooperative patents, overseas technology licensing, and clinical collaboration, on the quantity and quality of patents. Future research can integrate multi-source and cross-national data to establish a more comprehensive evaluation system for domestic IURH cooperation in China. Also, this study classifies innovation actors primarily according to the organizational identities recorded in patent application data and does not further distinguish the complex overlaps between actual innovation performers and formal patent applicants. Future research can use patent data or information on research projects to further identify the primary entity of innovation.
2. There are limitations in the selection of influencing factors. In this paper, 11 explanatory variables are selected from three dimensions, namely, economic development, residents’ income, and scientific and technological innovation. However, the factors affecting the provincial network status are far more numerous than these. Factors such as the level of international collaboration, the policy environment, the industrial foundation, talent reserves, and regional innovation culture may also play important roles. Subsequent research can attempt to introduce methods such as qualitative comparative analysis for further in-depth investigation.
3. The depth of analysis at the medical institution level needs to be strengthened. This paper identifies the evolutionary role of medical institutions in the network at the patent applicant level, but it fails to explore the differences between medical institutions of different types and levels in depth. Future research can combine case analysis or questionnaire surveys to further refine the understanding of the innovative behavior of medical institutions.

## Data Availability

The raw data supporting the conclusions of this article will be made available by the authors, without undue reservation.

## Footnotes

1
http://www.aimd.org.cn/newsinfo/5295207.html

2
https://www.giiresearch.com/report/psm1909326-ai-enabled-medical-devices-market-size-share.html

3
https://www.gov.cn/zhengce/content/2018-04/28/content_5286645.htm

4
https://www.gov.cn/zhengce/zhengceku/2022-08/12/content_5705154.htm

5
https://kw.beijing.gov.cn/zwgk/zcwj/202507/t20250703_4141266.html
